# Supplementary material for: Effect of sodium hydroxide dosage on strength development in cement-fly ash mortars: Experimental and ANN-based prediction
Source: PLoS One. 2026 Jan 21;21(1):e0331706. doi: 10.1371/journal.pone.0331706 (PMC12822939; doi:10.1371/journal.pone.0331706)
Supplement: Attachment — Submitted filename: Comments.docx [file pone.0331706.s001.docx]

I appreciate your valuable effort and contribution to this research study, which provides significant insights into the field. there are some comments and suggestions outlined below that need to be addressed for further refinement and improvement. I recommend considering these points to enhance the study's clarity and overall quality.

- 157 lines: Water used was tap water; (revise the statement)
- 158 lines: Alkali used was sodium hydroxide produced by Tianjin FengChuan chemical
- 159 lines: reagent Technology Co., Ltd., with NaOH content≥96.0% and carbonate ≤1.5%. (Please explain the Sodium silicate combined with Sodium hydroxide.
- The study was conducted on an alkali-activated material (AAM) that was formulated based on the following principles:

a. Aluminum silicate precursor: fly ash;

b. Alkaline activator: sodium hydroxide;

c. Fine aggregate: sand.

Remove this statement The above statement it is duplicate and already explains the materials from the material section

- GBT-17671 procedure reference is required for this method standard.
- Reduce the bullet and number for the method section and make it a paragraph instead of bullets. And please check all sentences with punctuation marks.
- After heat curing, all samples were maintained under laboratory conditions (temperature of 20±2°C and relative humidity of 90%) until testing. (Justify the statement using the Standard Code.)
- The test was performed at a loading rate of 0.2 MPa/min, a compressive strength sensitivity of 50 kN, a loading rate of 0.01 MPa/min, and a flexural strength sensitivity of 2 kN. (The loading rate needs to be cited with reference. In addition, please all statements from the methodology need to be cited.
- Mpa should be MPa, and check all errors in the manuscript.
- 238 lines: For FA mortars containing sodium hydroxide (N #)???
- Line 270, 283, 288: Each paragraph started with numbering, please remove numbers, (This is required for the entire manuscript.
- The title of research needs to be modified from (The Effect of an Alkali Activator on the Properties of Fly Ash-Based Mortar and Neural Network Prediction) to (The Effect of NaOH Percentages on Properties Cement Modified Fly Ash and Neural Network Prediction). The mixture contains Cement and percentages of fly ash; thus, it is not an alkali activator mortar, it is the cement mortar modified fly ash, containing NaOH concentration that why the title has to be clear.
- The discussion section combines with the result section, for instance: The compressive strength section explains the result and discussion in the same section.
- Resolution of Figures 1, and 7 have to be increased.
- Add recommendations and future perspectives.
