# Supplementary material for: Effect of sodium hydroxide dosage on strength development in cement-fly ash mortars: Experimental and ANN-based prediction
Source: PLoS One. 2026 Jan 21;21(1):e0331706. doi: 10.1371/journal.pone.0331706 (PMC12822939; doi:10.1371/journal.pone.0331706)
Supplement: Attachment — Submitted filename: Response to Reviewers-0606.docx [file pone.0331706.s006.docx]

**Comment No.1:** The current title lacks clarity and could be improved to more accurately reflect the study’s scope. A suggested revision is: 'Effect of Sodium Hydroxide Dosage on Strength Development in Cement-Fly Ash Mortars: Experimental and ANN-Based Prediction.' This version better communicates the dual focus on experimental analysis and neural network modeling.

Reply： Revised

**Comment No.2**: While the integration of Artificial Neural Networks (ANN) with alkali-activated materials is well-established in the literature, the manuscript would benefit from a clearer explanation of its original contribution. Specifically, you should articulate how the selected NaOH dosage levels provide new insight into material behavior, and what distinguishes the applied neural network model from previous studies.

Reply： How the dosage levels of NaOH can provide new insights into material behavior is discussed in lines 75-85. The description of the neural network model can be found in lines 111-134.

**Comment No.3:** The methodology section related to the ANN lacks sufficient elaboration. Further clarification is needed on the selection criteria for network parameters such as the number of neurons, layers, and the use of the trainlm training function. The manuscript would also be strengthened by describing the use of training and validation datasets and how model performance was assessed. In addition, the inclusion of standard performance metrics, such as root mean square error (RMSE) or mean absolute error (MAE)would help evaluate the model's effectiveness in predicting outcomes.

Reply： Modified in 2.2

**Comment No.4:** The sensitivity analysis is currently limited in scope and would benefit from deeper exploration. Additionally, the manuscript does not discuss the uncertainty associated with ANN predictions. Incorporating statistical measures such as confidence intervals or prediction bounds would enhance the robustness of the modeling results. I also recommended that you can demonstrate the model’s performance on data not used during training to assess generalization.

Reply： Revised, Due to the limited space of this paper, sensitivity analysis, uncertainty analysis, confidence interval and other methods were conducted. However, the details were only described in the text and will be added in the next paper.

**Comment No.5**: The manuscript contains grammatical inconsistencies and some technically imprecise expressions (e.g., “NaOH content≥96.0%”, “alkali-added mortar”, “colloidal sand”). A comprehensive language review by a proficient English editor is recommended to improve overall clarity and ensure terminological consistency throughout the paper.

Reply： Revised. NaOH content is no less than 96.0%. colloidal sand is changed to colloidal sand mixture.

**Comment No.6:**Several figures (notably Figures 3, 5, and 8) would benefit from enhanced resolution and clearer axis labeling. Table 2 is dense and may be more effective if divided or reformatted for readability. The scanning electron microscopy images (Figure 9) should include more descriptive captions and annotations to support the interpretations presented in the text.

**Reply：** The image is already the original image, Figure 9 has been added with the necessary comments, and Table 2 has added the title of the spread.

**Comment No.7:** The basis for choosing sodium hydroxide concentrations between 3% and 8% is not clearly stated. It would be helpful to indicate whether this selection was informed by previous literature or preliminary experimental work. Furthermore, the manuscript should provide a more detailed description of the control mix design used for comparative analysis.

**Reply：** Lines 172 to 173 of section 2.2 have been added, and on the basis of my preliminary experiments, the sodium hydroxide concentration of 3% to 8% has been selected.

**Comment No.8:**The term “colloidal sand” is non-standard and may lead to confusion. The authors are advised to define this term explicitly or adopt more conventional terminology. Moreover, consistency is needed in the usage of terms such as “geopolymer” and “alkali-activated material (AAM),” particularly when distinguishing between low- and high-calcium fly ash systems.

Reply： Revised. Colloidal sand is changed to colloidal sand mixture.

**Comment No.9**:The abstract includes repeated numerical values, which could be streamlined to improve its readability. Furthermore, the contribution of ANN to the study should be described earlier in the abstract to better represent the paper’s objectives.

**Reply：** The abstract has been rewritten as requested
