# Supplementary material for: Effect of sodium hydroxide dosage on strength development in cement-fly ash mortars: Experimental and ANN-based prediction
Source: PLoS One. 2026 Jan 21;21(1):e0331706. doi: 10.1371/journal.pone.0331706 (PMC12822939; doi:10.1371/journal.pone.0331706)
Supplement: Attachment — Submitted filename: Minor comments.docx [file pone.0331706.s005.docx]

1、 The commonly used molar concentration of sodium hydroxide in experimental conditions should not exceed 20M. Additionally, the ratio of sodium silicate solution to sodium hydroxide should not be greater than 1.0. (This limitation must be supported with appropriate references from recent and credible sources.)

**Reply： The references (Alabduljabbar H 2020 and LIU Fei peng, et al. 2022) have been added.**

2、 Several references cited in the manuscript are dated prior to 2022. The authors are advised to update the literature review by including more recent references published from 2022 onwards.

**Reply： Seven references published after 2022 have been updated.**

3、 Please check all abbreviations used throughout the manuscript. For example, clarify whether "P.O" or "O.P" is correct and ensure consistent use of abbreviations.

**Reply： All instances of “P.O” in Section 2.1 Materials have been uniformly changed to “OPC.”**

4、In Section 2.2 on Density, the method described must comply with a recognized standard. The authors should clearly state which standard was followed and provide a proper citation.

**Reply：GB/T 1596-2017 has been incorporated into the text and added to the reference list as entry 9.**

5、 Each paragraph in the manuscript must include relevant references to support the statements made. Proper citation is essential for maintaining scientific credibility.

**Reply： The revisions have been reviewed and confirmed.**
